# Building meaningful partnerships: engaging and co-developing a patient advisory council to inform the implementation of person-centred quality indicators in Alberta primary care

**DOI:** 10.1186/s40900-026-00970-w

**Published:** 2026-08-28

**Authors:** Matthew Luzentales-Simpson, Ingrid Nielssen, Fakhriyya Aghabayli, Sadia Ahmed, Kalpana Thapa Bajgain, Ifrah Anjum, Veronika Kiryanova, Safa Ahmed, Kim Giroux, D’Arcy Duquette, Eleanor Benterud, Paul Fairie, Maria J. Santana

**Affiliations:** 1 Department of Community Health Sciences, Cumming School of Medicine, University of Calgary, Calgary, AB Canada; 2 Alberta Strategy for Patient-Oriented Research Support Unit, Calgary, AB Canada; 3 Person-Centred Quality Indicators Patient Advisory Council, Calgary, AB Canada; 4 Department of Pediatrics, Cumming School of Medicine, University of Calgary, Calgary, AB Canada

**Keywords:** Person-centered quality indicators, Primary care, Patient advisory council, Patient engagement, Patient-oriented research

## Abstract

**Background:**

Effective primary care improves patient outcomes by providing comprehensive, continuous, and person-centred care. The Patient’s Medical Home vision positions primary care as the central entry point to high-quality health care in Canada. In Alberta, Person-centred Care Quality Indicators (PC-QIs) were developed with patients and health system stakeholders, are being implemented across the province to assess and report patient experiences in primary care. To ensure that patients are central to this work, a PC-QI patient Advisory Council (PAC) was created.

**Methods:**

The PC-QI PAC was formed in 2024 to support the provincial implementation of PC-QIs in primary care. The council included ten total members, eight patient research partners and two academic researchers, who co-developed a living Terms of Reference to guide the PAC’s operations. Monthly PAC meetings (later adapted to quarterly meetings with monthly newsletters) and working group meetings supported engagement. The patient engagement was evaluated twice using the Patient Engagement in Research Scale.

**Results:**

The PC-QI PAC contributed to the development of the Terms of Reference, research participant recruitment strategies, co-development of study materials, knowledge translation activities, and research dissemination. Members also co-authored abstracts, posters, and manuscripts, and participated in academic conferences and meetings. Key facilitators of engagement included the co-developed Terms of References, transparent communication, flexible participation in working groups, appropriate compensation, and leadership roles for patient co-chairs. Identified challenges included maintaining clarity of roles, sustaining engagement in virtual settings, and ensuring that members’ contributions to the broader project were clearly communicated and understood.

**Conclusion:**

By managing expectations, co-developing operations, and thoughtfully sharing information, the research team built meaningful, trustworthy, and long-lasting partnerships with patient partners, which benefited every team member and the research project. This study aims to describe and analyze the co-development and work of the PC-QI PAC to identify factors that shape meaningful patient engagement in the design and implementation of research in primary care, contributing to the limited evidence on this topic.

**Supplementary Information:**

The online version contains supplementary material available at https://doi.org/10.1186/s40900-026-00970-w.

## Abstract (plain language summary)

When people have a regular doctor or clinic, they are more likely to stay healthy and get the healthcare they need. In Alberta, Canada, Person-centred Care Quality Indicators (PC-QIs) are being introduced to assess and report patient experiences in primary care. To ensure patient voices are included in this process, a Patient Advisory Council (PAC) has been established. This paper examines how the council was created, and how it helps patients be meaningfully involved. The PC-QI PAC was formed in 2024 and includes patients with diverse lived health experience working together with researchers. The group met regularly, and adapted since 2024 to help members collaborate according to their capacity and interest. PAC members helped develop and update guiding documents, and co-created research materials for the project. They also co-presented this work at academic conferences. Patient engagement was continuously evaluated using anonymous feedback tools. Many things helped to make engagement on the PAC meaningful. Clear guiding documents and honest communication were critical, as were ensuring patients had flexible ways to participate, including leadership roles for patient partners. Providing compensation to patient partners was also important. Some challenges that were encountered include ensuring role clarity, maintaining member engagement in virtual meetings, and clearly communicating and recognizing patient partners’ contributions. Overall, this paper describes how meaningful partnerships between researchers and patient partners can improve research and health care. The lessons learned from the experience of the PC-QI PAC offers practical guidance for future projects aiming to involve patients in research and healthcare improvement.

**Supplementary Information:**

The online version contains supplementary material available at https://doi.org/10.1186/s40900-026-00970-w.

## Background

The World Health Organization (WHO) has described primary health care as care that addresses the broad determinants of health, including physical, mental, and social wellbeing. Accessible and timely primary health care has been described as the most efficient, cost-effective, equitable, and inclusive way to enhance people’s overall health [1].

The Patient’s Medical Home (PMH) is a vision for Canadian primary care to be an entry point for Canadians to access high-quality and timely health care services [2]. The PMH provides Canadians with access to adaptable, continuous, person-centred care (PCC) that responds to patients’ preferences and expectations and empowers them to actively participate in decisions about their health and health care [3, 4]. In addition to the care benefits, efficient access to high-quality primary care is associated with lower use of emergency services and reduced hospital admissions compared with individuals without such access [5, 6]. However, achieving this vision requires ways to measure whether care is truly person-centred, and aligned with the principles of the PMH, in practice.

In Alberta, Canada, limited access to primary care exacerbates inequities in health care access, experiences, and outcomes, particularly among equity-deserving populations, and negatively affects patient satisfaction and overall health outcomes [5, 7]. A successful and sustainable PMH dedicates resources to performance measurement, continuous quality improvement, and integrated research [2]. In this context, continuous and accurate collection and evaluation of the patient’s health care experiences is essential for improving the quality and continuity of care in health care settings, including primary care [8, 9]. The Alberta Government has established the “Modernizing Alberta’s Primary Health Care System (MAPS)” initiative to improve this situation and one of the proposed recommendations to support this initiative was to “promote measurement, evaluation, and quality improvement” to ensure effective implementation of health care initiatives [10]. This initiative aligns with the growing global trend to include patient centred outcomes and experiences in performance measurement sets in various care settings at different levels of the healthcare system [11–14]. Despite this, a recently published scoping review describes how the systematic collection and use of Patient-Reported Experience Measures (PREMs) in primary care is unevenly distributed at the macro and meso levels [15].

Person-Centered Quality Indicators (PC-QIs) are healthcare performance measurement tools which were developed with patient and family input, using a modified Delphi Consensus process, in Canada [9, 16–19]. PC-QIs measure the person-centeredness of healthcare delivery, which, when integrated into quality improvement processes, gives healthcare systems and providers the opportunity to improve their delivery of PCC. PC-QIs were designed to measure the healthcare experience from the perspective of the patient, meaning that the reporting of PC-QIs require the systematic collection of patient experiences to be implemented into the care setting where they are being used [9]. To ensure that PREMs and the PC-QIs are sustainably implemented with a focus on improving the patient experience, it is critical that we continue to engage with patients throughout the implementation and evaluation.

Considering the core principles of patient-centered care, including patients as partners in research has become integral, with patient-oriented research recognizing the importance of meaningful engagement with patient partners based on the principles of inclusiveness, support, mutual respect and co-building [20]. Patient advisory councils (PACs) are an effective approach to integrating patient engagement into patient-oriented research studies and activities [21–23].

This study contributes to the limited literature on PACs in implementation research by providing a co-developed, operational model for sustained patient engagement. This gap is particularly relevant in the context of implementing tools such as PC-QIs, where sustained and meaningful patient engagement is critical to successful implementation. This study aims to describe the co-development and operation of the aptly named PC-QI Patient Advisory Council (PC-QI PAC) to identify factors that shape meaningful patient engagement in the design and implementation of research in primary care.

## Methods

This patient-oriented research study aims to describe the co-development and work of the PC-QI PAC to operationalize patient engagement in the context of our program of research. Here we will describe how we engaged with patient partners early in the formation of the PC-QI PAC, and we will share recommendations that align with the Canadian Institutes for Health Research (CIHR) Patient Engagement Framework. Patient partner perspectives were identified using the Patient Engagement In Research Scale (PEIRS-22). The following sections describe PAC recruitment and composition, engagement structures, and evaluation of patient engagement.

### Setting

#### PAC member recruitment

Patient research partners (PRP) for the PC-QI PAC were recruited in early 2024 with support from the Alberta Strategy for Patient Oriented Research Unit (AbSPORU) Patient Engagement Team through their online platform Albertans4HealthResearch [24] [Supplementary Mat 1 PC-QI Recruitment post AB4HR.ca]. As the work of the PC-QI PAC is focused on a primary care implementation project, PRP recruitment was open to the general public, reflecting the access principles of Canada’s universal primary care services.

#### PAC membership & composition

Since April 2024 the PC-QI PAC has maintained a membership of ten members. Eight members are patient research partners (PRP) representing a diversity of gender, age, lived health and research experience, geographical backgrounds (rural and urban) and professional backgrounds from student to retiree. Two members are academic researchers (AR), and there are additional research support staff that are looped into PAC activities as needed. The PAC has two co-chairs, one a PRP and the other an AR.

### Terms of reference

**Fig. 1:**
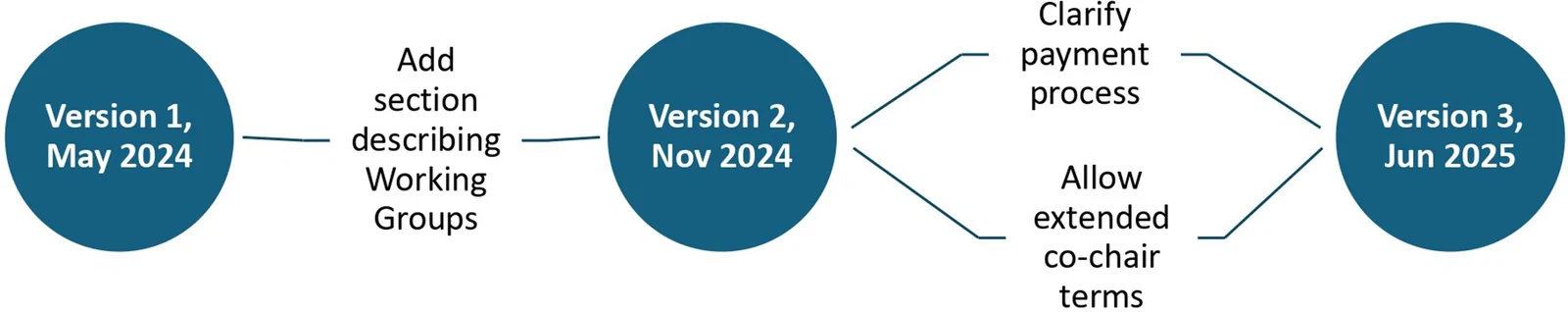
Terms of reference: document evolution

A co-developed living Terms of Reference document guides the operations of the PAC including guidelines for membership, meetings, withdrawing, and recruitment and onboarding of new members). To date this document has been revised three times. (See Fig. 1. Terms of Reference: document evolution) Using the AbSPORU Patient Engagement Team’s Terms of Reference (ToR) Template, the PC-QI PAC ToR was co-developed at the May and June 2024 PAC meetings as a guiding document [Supplementary Mat 2 Terms of References] where the PC-QI PAC’s purposes were established as follows:

- Advise on strategy for participant recruitment, knowledge translation (KT), and PAC operations.
- Provide insights and feedback to the overall study and PAC operations.
- Co-develop deliverables like KT materials, interview guides, and manuscripts.
- Co-conduct focus groups with survey participants, and evaluations of this PAC.
- Connect with other primary care stakeholders (ex. other patient advisory groups).

In addition, the ToR also described the expectations for all members of the PAC, including those of the patient co-chair, the research team members, and general members. The ToR also described the compensation process and outlined a meeting schedule for the first year of PAC operations. The ToR has since been iterated upon, and changes are described briefly in Fig. 1.

### Communication

At the establishment of the PC-QI PAC, all PAC members discussed the tools which would be used to support information sharing and communication. It was determined by the PAC that a shared Google Drive would be created to support collaboration and document sharing across the team. Shared documents include co-authored research outputs like conference poster presentations, research abstracts, recruitment posters, and manuscripts; operational documents like the ToR, compensation tracking sheets, meeting agendas, notes, and recording links; and working together documents which are often in the form of discussion notes from Working Group Meetings. In July 2024, an anonymous feedback form was created to provide PRPs with an additional, confidential mechanism to share feedback, without concerns for negative consequences. The anonymous feedback form has been used to collect feedback on specific PAC activities (e.g., conference poster presentations, monthly newsletters) and general experiences within the PAC, including both structured and open-ended responses.

**Fig. 2:**
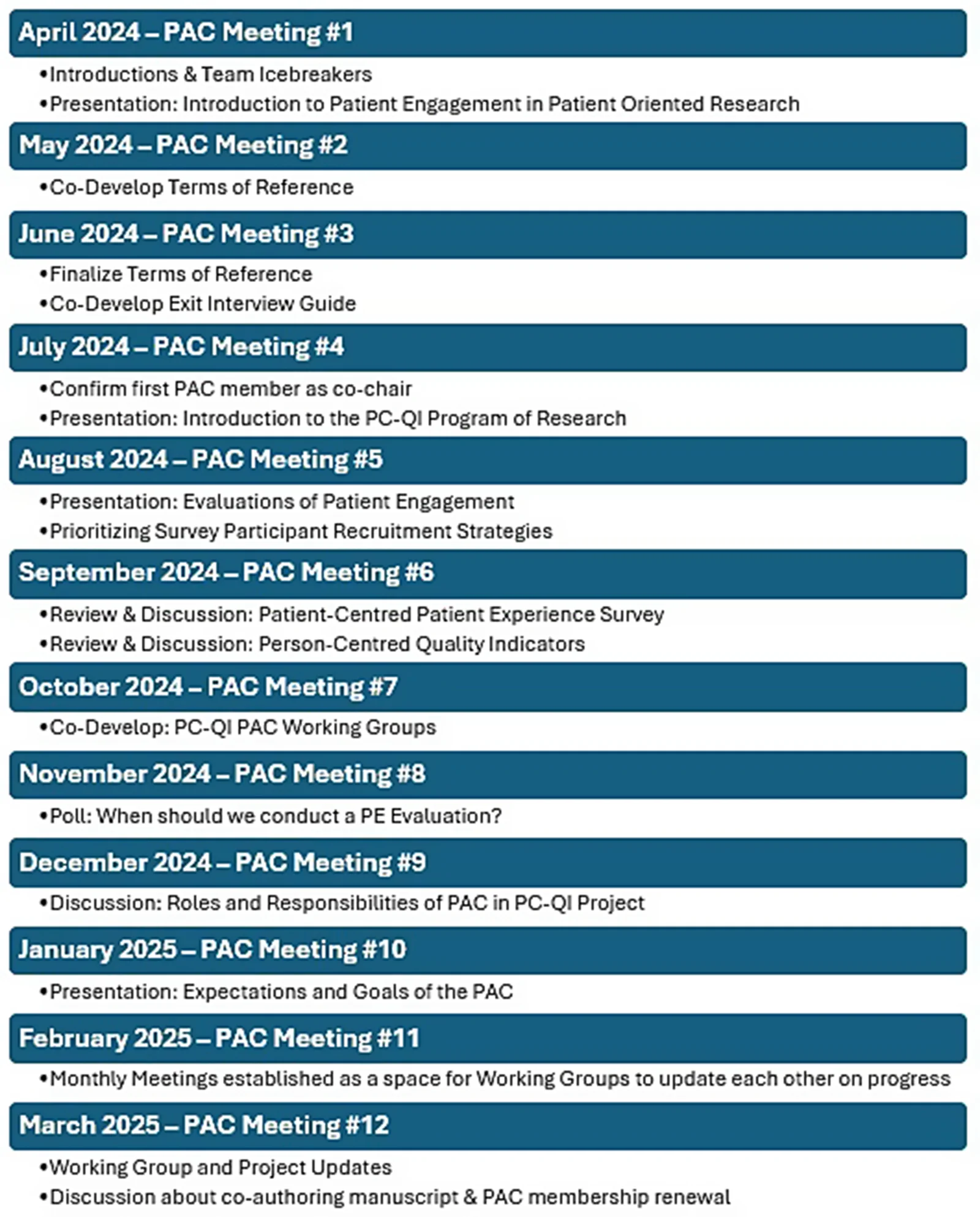
PC-QI PAC meetings

The PC-QI PAC had monthly meetings to discuss updates on its work and the research project. Figure 2 briefly describes the purpose and sequence of monthly meetings from April 2024 to April 2025. (See Fig. 2. PC-QI PAC meetings). Understanding PRPs need for flexibility in attendance, PC-QI PAC meetings were recorded and shared with all PAC members. and a clause was described in the ToR to describe the expectations for PAC members should they be unable to attend meetings [Supplementary Mat 2 Terms of Reference], essentially asking PRPs to take it upon themselves to discuss how to improve their engagement and attendance with co-chairs, if possible.

At the monthly meeting in September 2025, it was decided the PAC would transition to holding quarterly meetings with monthly newsletters to update members as there were not sufficient engagement opportunities or discussion to be had at monthly meetings. An agenda for meetings was developed by co-chairs and shared with all PAC members before the meeting date. Meeting notes were shared with all PAC members about one week after the meeting, and they included a hyperlink and passcode to the recording of the meeting. Starting in September 2025, a newsletter was created and updated by an AR co-chair, and shared with all PAC members on a monthly basis to ensure PAC members have regular, yet brief, updates on the project. The newsletter also includes regular reminders and hyperlinks to support PAC members’ engagement in research activities. (Supplementary Mat 3. November PAC Newsletter)

### Working groups

To support workflow and engagement, seven task-oriented working groups with two to five PRPs were established: (1) Knowledge Translation; (2) Co-Creation of the PC-QI PAC; (3) Patient Engagement Evaluation; (4) PC-QI Training Program; (5) Project Promotion; (6) Grant Funding; (7) PREM Evaluation. PRPs were invited to attend and collaborate on the specific activities of the working groups for which they were interested and available.

### Compensation and reimbursement

PRPs were offered compensation at the suggested rate in the AbSPORU Patient Partner Compensation guidelines of (CAD) $25 per hour. A tracking sheet describing activities and recommended billable hours was established, housed in the shared drive and updated regularly by the ARs. PRPs submitted their recorded hours twice a year using a submission form. In addition to their hours, they also shared their preferred method of payment. Compensation for the PAC was paid in the form of honoraria, or gift cards.

In addition to compensation, all PAC members were either reimbursed for any travel and attendance related costs for their participation in conferences, or these were paid up front whenever possible.

### Patient engagement evaluation

To assess the PC-QI PAC member’s experience working together, an AbSPORU Patient Engagement team member conducted two patient engagement evaluations. The first evaluation was conducted in July 2025, and the second evaluation in February 2026. The PEIRS-22 tool was used to assess the experience of patient partners working in the PAC by asking 22 questions across 8 themes which make up meaningful patient engagement [25, 26]. Patient Engagement Working Group members reviewed the results of the evaluation with PAC members, including them in the interpretation of the data. Evaluation results were then also reviewed collaboratively with PAC members and used to inform ongoing improvements to the engagement processes. Discussions and recommendations generated from the interpretation of the baseline survey data are included in the results of this study. The results comparing the different timepoints of the patient engagement evaluation will be published separately.

## Results

### PAC contributions towards patient engagement

Foundational knowledge surrounding the principles patient-oriented research and patient engagement was introduced at the inaugural PC-QI PAC meeting in April 2024, presented by the AbSPORU Patient Engagement Team. Establishing a common understanding of these principles, guided by the CIHR Patient Engagement Framework, helped to establish baseline expectations for members. The ToR, as described in the Methods, was revised twice (Fig. 1). The first revision was made after a thorough discussion between all PAC members about their roles and expectations starting in October 2024 (Fig. 2), and led to the formation of Working Groups within the PAC, allowing PRPs to contribute based on their interests and capacity. For example, the PC-QI Training Program Working Group worked together closely and frequently to co-develop a Training Program for primary care providers which describes how clinicians might work with PC-QIs in practice. Whereas the Knowledge Translation Working Group has primarily engaged in consistently reviewing and providing feedback on abstracts, posters, and other research materials, supporting the use of lay language and integrating the patient’s perspective into the dissemination of our work.

### PAC contributions to PC-QI research activities

**Fig. 3:**
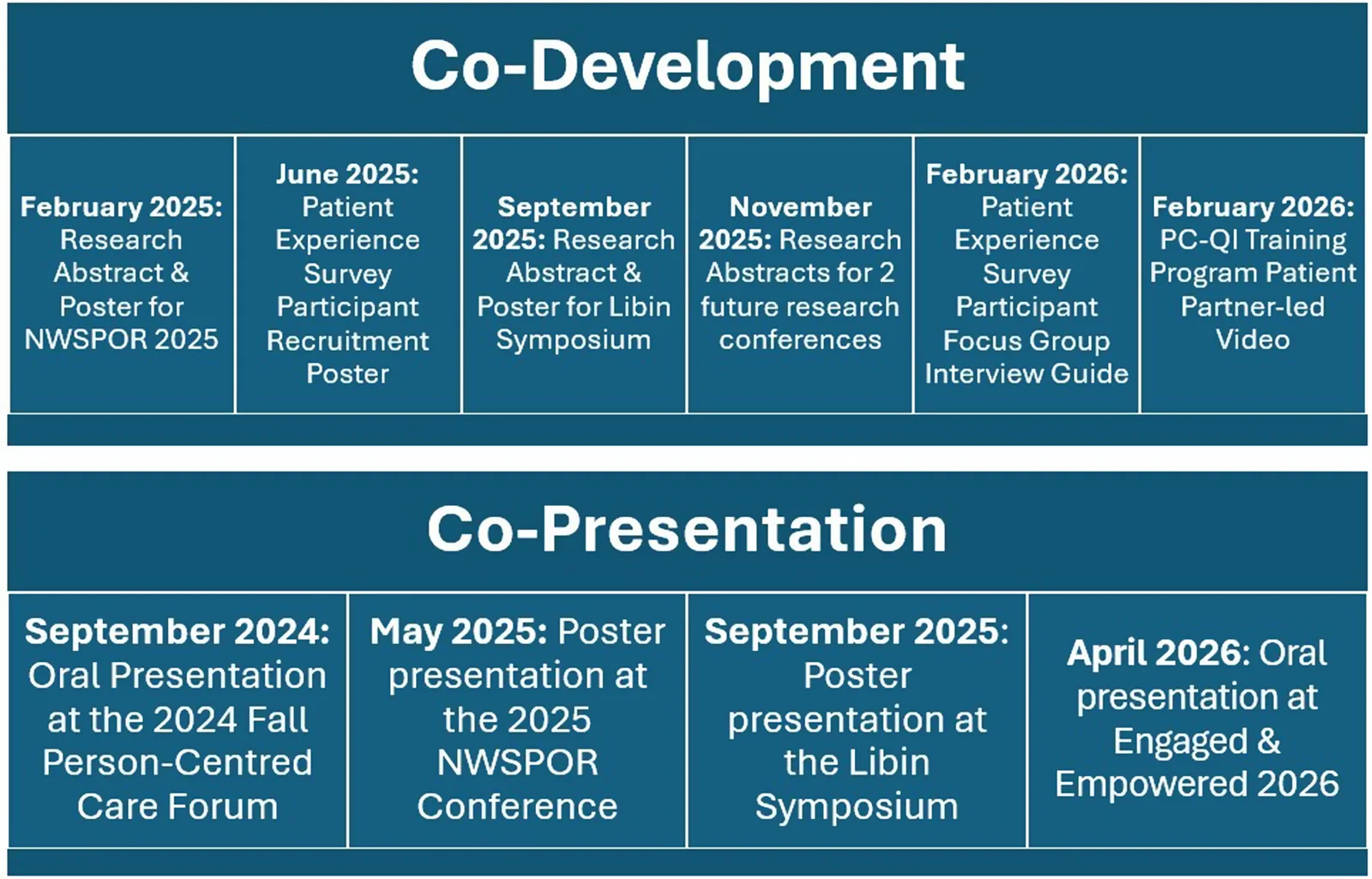
Overview of the PC-QI PAC contribution to research dissemination

PC-QI PAC members were actively engaged across multiple stages of the research process, including taking an active role in the dissemination of research projects findings through co-development and co-presentation of abstracts, posters, and oral presentations at conferences, writing plain English summaries such as for this manuscript. PRPs consistently provided feedback to the ARs about where to improve clarity using lay language and visual components in both oral and poster presentations. PRPs were invited to attend four local, Alberta-based research conference events since the group’s inception, giving PRPs the opportunity to co-present posters and oral presentations with ARs, in-person, while strengthening relationships between PAC members in attendance at these events. In addition to great engagement at local conferences, PAC members have provided key feedback on research abstracts which were accepted for national and international conferences, including but not limited to, the 2024 Canadian Association for Health Services and Policy Research Conference, the 2025 Canadian Outcomes Matter Conference, and the 2026 Gothenburg Centre for Person-Centred Care Conference. Figure 3 provides an overview of PAC members’ involvement in the co-development and co-presentation of research activities.

PAC members have also been involved in the co-development and review of other research products, including a recruitment poster and brochure, a PC-QI Training Program, and providing input on the patient-reported experience measure at the 6th PAC Meeting (See Fig. 2). Taken together, these activities reflect how patient partners were engaged throughout the research cycle, including in the study design, knowledge translation, dissemination, and co-production of research outputs.

### PAC findings

Collective insights were identified through group discussions, patient engagement evaluations, and ongoing feedback from PAC members while building the PAC. These insights were further refined with PAC members’ input, formally though our planned patient engagement evaluation, and informally through personal communications and conversation. These insights from the process of building and forming this PAC together are summarized in Table 1, organized according to the four guiding principles of the CIHR Patient Engagement Framework: Inclusiveness, Support, Mutual Respect, and Co-Build.

**Table 1:** List of insights surrounding the operationalization of each fundamental principle of the CIHR Patient Engagement Framework, in the PC-QI PAC

CIHR Patient Engagement Framework Principle	Collective Insights
Inclusiveness	Reserve time for relationship building, through icebreakers, casual conversation during and around meetings, and at opportunities to work together in-person
	When explaining complex concepts, use visuals and lay language wherever possible
	Working Groups can support working with PAC members who have varied levels of engagement, and diverse interests and expertise
	Provide a safe space for PAC members to discuss expectations, and to speak openly and honestly with one another
Support	Ensure that compensation practices are clearly defined and communicated, including the specific responsibilities of PAC members, co-chairs, and research team members
	Research teams should provide adequate time and resources to allow PAC members the space to learn and an opportunity to identify what they need/want to learn to participate fully
	Consult with PAC members on user-friendly software to support working together
	Consult with PAC members on their preferred communication method for different types of communications
Mutual Respect	Set specific expectations for all PAC members earlier, rather than later
	Transparency about changes to project progress from the research team to PRPs can build trust and promote open communication
	Review patient engagement principles to set foundational knowledge & expectations in an introductory meeting
	Clearly set realistic expectations for PAC members regarding their role in the project, time commitment of each role, and the compensation or reimbursement being offered for each role
Co-Build	Ensure members have a voice in the development of PAC operations, through the establishment of a living Terms of Reference
	Plan to conduct a patient engagement evaluation, and allow PAC members to inform the evaluation
	Consider using Working Groups to generate co-built outputs, if you have a PAC with high membership
	Reserve space at regular PAC meetings for Working Groups to open discussions on the work they have done since the last meeting

### Inclusiveness

Inclusiveness is described as integrating a diversity of patient perspectives into research, ensuring that research is reflective of patient contributions [20]. In addition to an open recruitment method, we aimed to promote inclusiveness early in the formation of the PAC by reserving time at the inaugural PAC meeting for introductions and a brief icebreaker activity. This relationship building was identified as critical in our work, as we encourage casual conversations during and around meetings, as time allows, while aiming to come together at in-person opportunities throughout the year. When our research team works with the PAC, we also used lay language and visuals to support accessibility for all PAC members.

### Support

Support involved providing adequate resources and flexibility to patient partners, ensuring that they can contribute fully to research [20]. Support can be provided in a multitude of ways, including through financial compensation, and implies the creation of safe working environments. To ensure adequate financial support for patient partners, patient engagement was included in our initial project budgets. To ensure that financial support reaches the intended members in a manner that best suits them, we recommend co-developing compensation practices, where the responsibilities of all parties are defined. In the months spent co-building the PC-QI PAC, we also consulted with PAC members on what software we would use and had expressed where our team can provide learning support for use of new software that comes up in discussions. We also consulted with PAC members on their preferred communication method for different types of communications, to ensure that the time spent working together is used efficiently and respectfully. This discussion led to minor changes to our communication methods, such as using monthly e-mailed newsletters, with clear messaging instead of monthly virtual calls for project and timeline updates, while continuing to use virtual calls for Working Group discussions and quarterly project updates.

### Mutual respect

Mutual Respect is about acknowledging all parties’ expertise, including the multiple and diverse lived health care and health research experience and experiential knowledge that each PAC member held [20]. Collaboratively establishing expectations at the onset of working together can support mutual respect for each team member’s time and voice. Reviewing patient engagement principles in an introductory meeting, and throughout the co-building of the PAC, can set foundational knowledge for all members and help set expectations. Respect can also be reflected in regular and transparent communication with patient partners about project progress, or in other practices of open communication, for example meaningfully following up on team members’ questions, comments, and feedback to all the work being done together.

### Co-build

Co-building is about ensuring that patient partners and researchers work together early and throughout the research cycle to co-produce research priorities, best practices, study materials, and implement solutions to research gaps or obstacles [20]. To develop the skills and early processes for co-building, PAC members were included in the co-development of the living Terms of Reference document. Early engagement and involvement of PAC members in the planned patient engagement evaluation, and in future iterations of the Terms of Reference, emphasized how the principle of co-building would be a regular activity of the PC-QI PAC. Working Groups were also developed within the PC-QI PAC to support the co-building of various research project outputs, such as participant recruitment posters and informational brochures. By reserving space at regular PAC meetings for Working Groups to open discussions on the work they have done, we have also created a space where the co-built products and effort put forth by the Working Groups can be shared with and appreciated by other PAC members. Overall, meaningful engagement was supported by co-developed governance, flexible participation structures, and continuous feedback mechanisms, while challenges were primarily related to role clarity and communication.

## Discussion

While PACs have long been used as a facilitator for patient engagement across various settings, an American scoping review examining the use of Patient and Family Advisory Councils (PFACs) in US hospitals found that none of the studies described what qualities make their PFACs successful [27]. Literature describing the qualities that make PACs or PFACs successful are more common in health research settings [25, 28–33] there remains limited guidance on how to build and operationalize PACs within implementation-focused primary care research. In the context of implementing PC-QIs in Albertan primary care, building and forming a PAC helped us to ensure that patient perspectives are meaningfully integrated into the design and implementation of PREMs and PC-QIs. By engaging with patients using a PAC, we can integrate a range of diverse patient and caregiver experiences into the design of the implementation project that would be difficult to achieve through sporadic engagement or engagement of few PRPs. For example, considering the overall goal of PC-QI implementation is to provide supportive tools and systems to ultimately improve patient experiences in primary care, engaging people with lived experience in the design of the implementation is vital to ensure that the use of PC-QIs does not inadvertently interfere with patient satisfaction and care. The building and formation of this PAC can be used to contribute to practical recommendations for PAC development, organization, and sustainment for the long term [34]. Integrating these recommendations into the patient engagement process within other research projects will support PRPs in their role within PACs, while greatly benefiting research and implementation studies by ensuring inclusiveness of different perspectives, life experiences, and levels of engagement into all stages of research.

Our team recognized the group’s desire to ensure there was always at least one patient co-chair at any given time, ensuring that patient partners have a seat at the table with regards to decisions surrounding PAC affairs. This was explicitly reflected in Version 3 of the ToR, where the language around the co-chair position was changed to empower patient co-chairs to remain in the role for the length of time that best suits them, while keeping the door open for multiple patient co-chairs to sit on the PAC simultaneously. In the case of the PC-QI PAC, this allowed us to demonstrate our gratitude for the work that the inaugural patient co-chair had done by giving them the opportunity to continue serving the PAC in this leadership role until they were prepared to step away some months later. The change also demonstrated how members can take on more or fewer responsibilities depending on the time, energy, and interest they have available, indicating that patient engagement is not dichotomous (e.g., engaged or not engaged) [35]. Respect for their level of interest and time available. Another strategy our team deployed based on PAC member feedback and experience was to replace monthly meetings with an easy-to-read newsletter that includes actionable items in the email text. By adapting engagement structures to people’s changing capacity and preferences, we believe that we can improve communication and support structures in the PAC, furthering the notion that patient engagement is not necessarily dichotomous. Additionally, different formats (e.g., asynchronous updates vs. meetings) supported sustained engagement in different ways. Recently introduced asynchronous communications, via monthly newsletters for example, supported engagement by reminding PAC members of upcoming activities, supplemented with hyperlinks to the relevant supporting documents. This adaptation also improved the quality of engagement at meetings, where time can be used more efficiently to support engagement on decision-making requiring patient partner input, and interpreting data, for example.

Mutual respect has been described by others as a key indicator of an effective PAC, that takes the form of a safe working environment, honest and transparent communication, and support [31–33]. The work of engagement-capable environments describes three main pillars of engagement [36]. The first pillar of enlisting and preparing patients aligns with the process we took to recruit PRPs, as described in the methods. led to our research team having short phone calls and Zoom meetings with potential PRPs, as there were more people interested than the PAC had spots for. In doing so, we were able to recruit PRPs with varying degrees of research participation and partnership, which has been described as a powerful way of supporting new PRPs to gain the skills to understand what meaningful engagement looks like [37]. The second pillar of engaging staff to involve patients and to recognize power and identity, was primarily built together through the early co-building that was done in the PAC. This required significant reflection on our early practices of co-building, recognizing there was room to better engage and empower PRPs in the design and implementation of our project. Reflection often came in the form of honest conversations within a safe environment between a PRP and the co-chairs, and sometimes through the anonymous feedback form that was shared with the PAC. The third pillar of ensuring leadership support and focus was already well engrained in the ARs and support staff involved in setting up the PAC, through years of experience in patient-oriented research in Canada. In additional to the established culture that respects patients’ needs and lived experience, there were also tangible and specific supports they offered to the PAC including organizing the first foundational PAC meeting in April 2024, providing working templates to support the development of the ToR and the tracking of PAC activities, and conducting the formal evaluation of patient engagement using the PEIRS-22 tool. The work of the PC-QI PAC to improve as an engagement-capable environment has resulted in meaningful cooperation on several different tasks within the research project, including the co-presentation of our research at academic conferences, and in the co-development of research products (e.g. participant recruitment posters, focus group interview guides). The building of this PAC has provided a unique opportunity for all members to learn from one another and to co-build a productive and safe environment within the team to help with identifying problems and gaps, setting priorities for the research project, and working together on solutions. Therefore, emphasis on an engagement-capable environment that prioritizes mutual respect and improved two-way communication encouraged our team to have honest conversations about the level of engagement, and the process by which engagement is occurring in different areas pertaining to the program of research.

### Challenges

There was significant work involved in keeping accurate and timely records of PAC members’ hours worked across various activities and working groups. This challenge, combined with a lack of clarity surrounding timelines for compensation in the ToR, resulted in delays to the compensation process initially. We are working to improve this through the third version of the ToR which clarifies the expectations, combined with regular reminders via monthly newsletters.

The virtual nature of the PAC meetings presented a challenge to keep PAC members engaged in all discussions. Working together in-person, primarily through co-presentations at conferences, has been a more engaging experience for PAC members. Though working together in-person has been mostly positive, some PAC members mentioned that they feel their role in this research project mostly included participating in abstract writing, manuscript reviews and contributions, and developing posters and presentations for conferences. They felt that they did not contribute to the project itself, meaning the implementation of PCQIs at primary care clinics. In reality, PAC members contributed to the project in significant ways, particularly through their participation in the development of the abovementioned research products, and by giving feedback at different stages of the research cycle. We think this, and the other challenges described above, can be attributed to unclear communication between the research team members and PAC members about the progress of the project, and contributions of the working groups. Updated communications processes and evaluating patient engagement in the PC-QI PAC using the PEIRS-22 tool will likely improve our understanding of these challenges and how best to tackle them.

### Next steps

We are conducting the first follow-up PE evaluation with the PC-QI PAC, using the modified PEIRS-22 tool in early 2026, and will be planning to complete a second follow-up PE evaluation for approximately one year from now. We will compare the results of these evaluations to the baseline that we collected in August 2025, and we plan to work together to identify and implement strategies to continue to improve our engagement of PAC members.

## Conclusion

By co-developing operations, engaging with patients throughout the entire research cycle, and through adaptive governance driven by patient co-leadership, the research team built meaningful, trustworthy, and long-lasting partnerships with patient partners, which benefited every team member and the research project. We provided evidence of how we used the CIHR PE framework in our PAC to conduct effective and sustained patient engagement. We also described how we informal and formal feedback mechanisms to identify where there may be opportunities for improvements in the patient engagement, promoting flexibility, authentic inclusivity, and respect for others.

## Supplementary Information

Below is the link to the electronic supplementary material.


Supplementary Material 1



Supplementary Material 2



Supplementary Material 3


## Data Availability

The data presented in this study is available from the PC-QI Patient Advisory Council, upon reasonable request in accordance with institutional policies and procedures.
