# Supplementary material for: Building meaningful partnerships: engaging and co-developing a patient advisory council to inform the implementation of person-centred quality indicators in Alberta primary care
Source: Res Involv Engagem. 2026 Aug 28;12:135. doi: 10.1186/s40900-026-00970-w (PMC13525678; doi:10.1186/s40900-026-00970-w)
Supplement: Supplementary file 1 [file 40900_2026_970_MOESM2_ESM.pdf]

AbSPORU
Alberta SPOR SUPPORT Unit

ALBERTANS 4 HEALTH RESEARCH

Improving the quality of the Patient Medical Home for patients: Implementing Person-Centred Quality Indicators (PC-QIs) in primary care in Alberta – Patient Advisory Council

University of Calgary

Background

This project aims to enhance the quality of The Patient's Medical Home (PMH) in Alberta by implementing Person-Centred Quality Indicators (PC-QIs) in primary care. These indicators, developed collaboratively with patients and healthcare providers, serve as evidence-based tools to ensure person-centred, interdisciplinary care. The project will involve working with primary care partners to co-design the PC-QI implementation, study influencing factors, create supportive and training materials, and evaluate changes in care quality at participating clinics.

The study will focus on seven academic clinics before expanding to family practices and rural primary care settings in the following years. Through collaboration with existing partners and a focus on health equity and inclusion, the project will support the provision of high-quality, patient-centred care in diverse Alberta populations. The findings will inform the widespread adoption of PC-QIs, ensuring sustained, person-centric care within The Patient's Medical Home model while aligning with provincial performance measurement and quality improvement initiatives. More about The Patient's Medical Home model here.

Roles and Responsibilities

We are seeking patient partners with lived experience accessing primary care/family doctor services in Alberta to join a patient advisory council to support the co-development of strategies and materials to train and inform clinical staff and patient participants on the project. In addition to their role on the advisory council, patient partners can expect to offered additional research and capacity building activities that are relevant to the scope of the project including to help develop strategies and materials to support participant recruitment, knowledge dissemination and other research and implementation activities. **Patient partners can expect the research team to provide a supportive and flexible environment that is friendly to people new to patient-oriented research.**

Time Commitment

The current expected time commitment for patient partners will be 1-year starting beginning of April 2024 to March 2025 with scheduled monthly online meetings (12 meetings) of about 1.5 hours each. There will be an additional 30 minutes allotted for preparing/closeout.
Additional opportunities for collaboration may be offered and patient partners are invited to join as they are interested and available.

Compensation/Reimbursement
Patient partners will be offered standard compensation as per the AbSPORU Patient Partner Appreciation Guidelines. See here.
This is anticipated to be an online activity however if any in-person meetings are scheduled patient partners will be offered reimbursement for any out-of-pocket expenses related to the engagement (i.e. parking, travel, etc.)

For more information or to express interest
Matthew Luzentales-Simpson
Matthew.luzenalessi@ucalgary.ca
403.689.8849

Or

Maria Santana
mjsantan@ucalgary.ca
