# Supplementary material for: Building meaningful partnerships: engaging and co-developing a patient advisory council to inform the implementation of person-centred quality indicators in Alberta primary care
Source: Res Involv Engagem. 2026 Aug 28;12:135. doi: 10.1186/s40900-026-00970-w (PMC13525678; doi:10.1186/s40900-026-00970-w)
Supplement: Supplementary file 2 [file 40900_2026_970_MOESM3_ESM.pdf]

PAC
PC-QI

Newsletter

NOVEMBER 2025

S	M	T	W	T	F	S
						1
2	3	4	5	6	7	8
9	10	11	12	13	14	15
16	17	18	19	20	21	22
23	24	25	26	27	28	29
30						

Canadian Outcomes Matter

MATT PRESENTED AT CANADIAN OUTCOMES MATTER 2025
(PRESENTATION CAN BE FOUND **HERE**)

LATEST PAC MEETING
MEETING MINUTES CAN BE FOUND **HERE**

DECEMBER 2025

S	M	T	W	T	F	S
	1	2	3	4	5	6
7	8	9	10	11	12	13
14	15	16	17	18	19	20
21	22	23	24	25	26	27
28	29	30	31			

WORKING GROUP MEETING TO DISCUSS PATIENT-LED VIDEO

NO PAC MEETINGS IN DECEMBER

HAPPY HOLIDAYS

Contact Matt & Fakhriyya if interested in:

- THE PCQI TRAINING WORKING GROUP IS INTERESTED IN INCORPORATING A PATIENT-LED VIDEO INTO THE PCQI TRAINING MODULES. PLEASE, **CONTACT US** IF YOU ARE INTERESTED IN WORKING ON THE VIDEO (SCRIPTING, STARRING, EDITING, INVOLVEMENT IN DECISION MAKING).
- IF INTERESTED IN CO-PRESENTING, CO-DEVELOPING ABSTRACTS OR PRESENTATION MATERIAL FOR FOLLOWING CONFERENCES, OR TO GIVE FEEDBACK:
  - **ALBERTA COLLEGE FOR FAMILY PHYSICIANS SUMMIT**
  - **CANADIAN ASSOCIATION FOR HEALTH SERVICES AND POLICY RESEARCH**

PATIENT-LED VIDEO

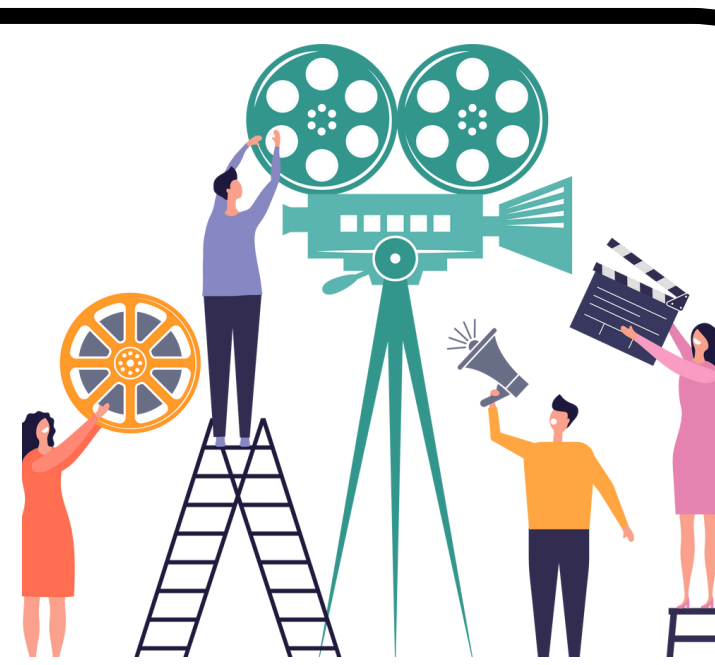

- WORKING GROUP MEETING (VOTE **HERE** TO FIND TIME THAT WORKS FOR EVERYONE)
- YOU CAN ACCESS VIDEO PLANNING DOCUMENT **HERE**

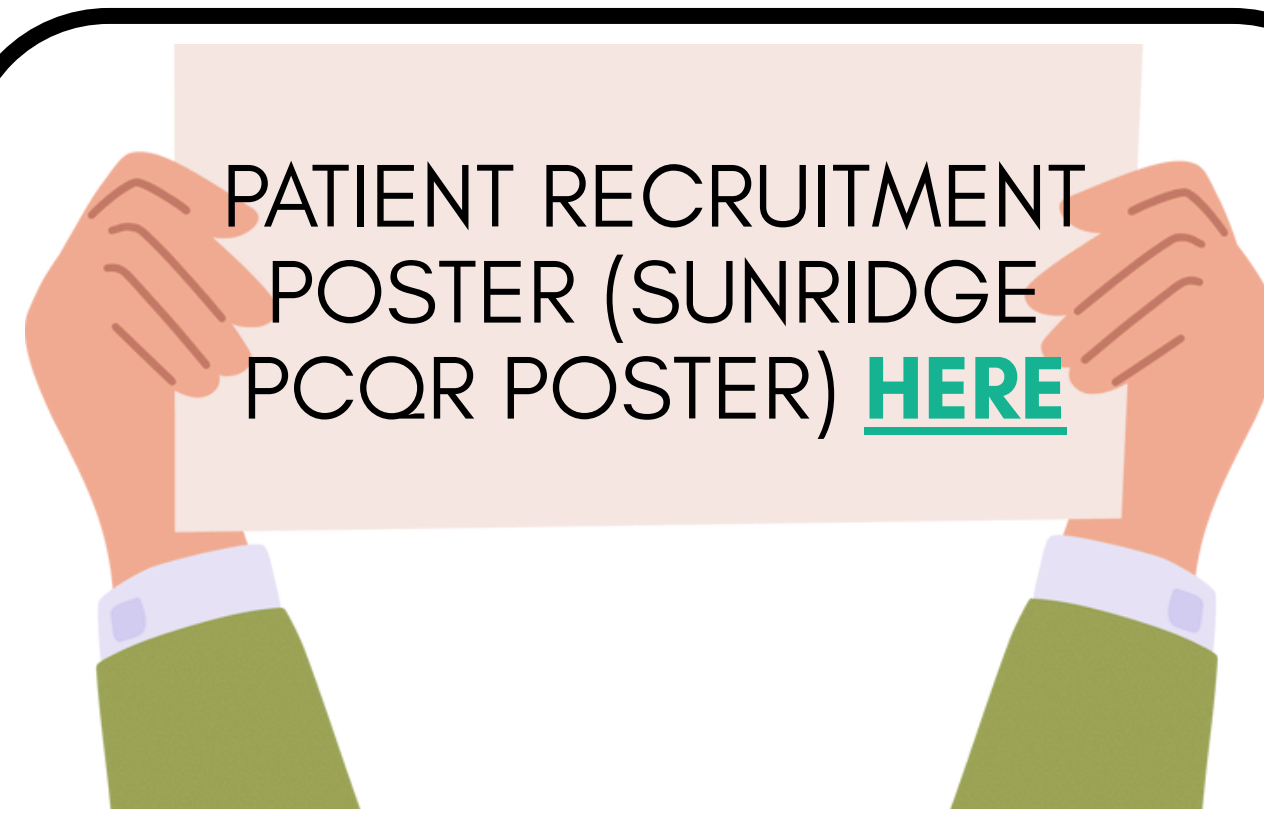

- WE NEED TO RECRUIT PATIENTS AT UNIVERSITY OF CALGARY CLINICS
- POSTER AND ADDITIONAL INFORMATION ON WEBSITE WILL BE DEVELOPED FOR THIS PURPOSE

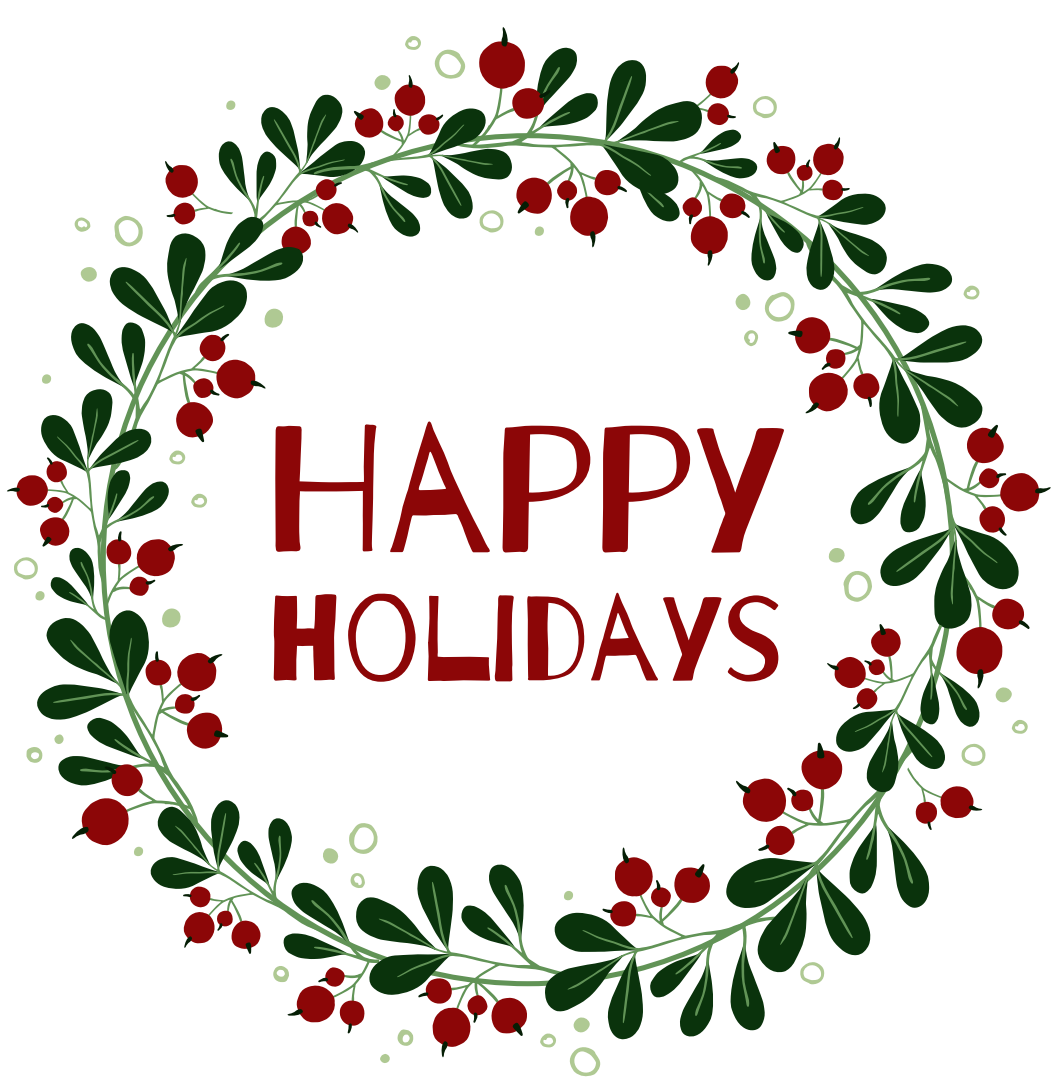

LINKS

Your Compensation Tracking Sheet

Previous Newsletters

Meeting minutes from November 24th PAC meeting
