# Supplementary material for: Building meaningful partnerships: engaging and co-developing a patient advisory council to inform the implementation of person-centred quality indicators in Alberta primary care
Source: Res Involv Engagem. 2026 Aug 28;12:135. doi: 10.1186/s40900-026-00970-w (PMC13525678; doi:10.1186/s40900-026-00970-w)
Supplement: Supplementary file 3 [file 40900_2026_970_MOESM1_ESM.docx]

**GRIPP2 Reporting Checklist – Short Form**

| **Section and topic** | **Item** | **Section where reported and details** |
| --- | --- | --- |
| 1. Aim | Report the aim of patient and public involvement (PPI) in the study. | Introduction (pq.6), Methods (pq.7 ) Patient engagement in this research was critical, to ensure patient perspectives continue to guide the research team in the implementation study. |
| 2. Methods | Provide a clear description of the methods used for PPI in the study. | Method (pq.7-10) Patient partners are included as study co-authors, indicating their level of involvement throughout the entirety of the study. |
| 3. Study Results | Outcomes – Report the results of PPI in the study, including both positive and negative outcomes | Results (pq.10-17) All patient partners and members of the PC-QI PAC played a key role in the interpretation of the data in the context of the co-development of the PAC. |
| 4. Discussion and Conclusions | Outcomes – Comment on the extent to which PPI influenced the study overall. Describe positive and negative effects. | Discussion (pq.17-19), Conclusions (pq.20) Patient research partners had a significant impact on the overall design, conduct, and writing of this study, since inception. Specific co-authors have their contributions detailed in the Author’s Contributions sections (pq.22). |
| 5. Reflections/ Critical Perspective | Comment critically on the study, reflecting on the things that went well and those that did not, so others can learn. | PAC findings (pq.12-17)  Positive outcome: Mutual trust and support between the research team and PAC.  Negative outcome: PAC members were sometimes confused about their role in the research project.  Area for improvement: Clear communication about every stage of the research project. |
